# Investigating LGBTQ affirmative attitudes and needs for better practice among Hungarian healthcare professionals

**DOI:** 10.1192/j.eurpsy.2024.96

**Published:** 2024-08-27

**Authors:** G. Vizin

**Affiliations:** University Budapest, Budapest, Hungary

## Abstract

**Abstract:**

Introduction: LGBTQ (lesbian, gay, bisexual, transgender, queer) people often do not seek health care and do not identify as LGBTQ people because of fear of judgment, stereotyping, and discrimination by health professionals. All of this is a particularly worrying phenomenon, because various mental difficulties, risky behaviors, and certain types of somatic and psychosomatic diseases may appear in a higher proportion among them.

**Objectives:**

Attitudes related to LGBTQ people were examined in several areas in Hungary. Most of our data comes from psychologists, however, a comprehensive examination of health professionals’ attitudes towards LGBTQ people has not yet been carried out.

**Methods:**

In a cross-sectional online survey, we ask healthcare professionals (medical doctors, nurses, other graduate healthcare professionals and medical university students) to fill out our questionnaire. The participants complete the Modern Homonegativity Scale and the Lesbian, Gay, Bisexual and Transgender Clinical Skills Development Scale.

**Results:**

We assume that the majority of Hungarian healthcare professionals have a neutral or positive attitude towards LGBTQ people, but they struggle with a significant lack of affirmative skills. We will present our results in detail in the presentation of the symposium.

**Conclusions:**

There is an urgent need to provide the appropriate affirmative knowledge material to Hungarian healthcare workers.

**Disclosure of Interest:**

None Declared

